# The role and mechanism of UPRmt in adipocytes

**DOI:** 10.1080/21623945.2026.2651605

**Published:** 2026-04-13

**Authors:** Hao Liu, Jie Chen, Dan-Qi Qiu, Miao-Wei Jiang, Hao-Qi Chen, Li Li, Shu-Qin Chen

**Affiliations:** Department of Endocrinology, Yuehu Campus, The First Affiliated Hospital of Ningbo University, Ningbo, People’s Republic of China

**Keywords:** Mitochondrial unfolded protein response, UPRmt, adipocyte, metabolism

## Abstract

Obesity is one of the most significant health challenges today, with its prevalence increasing rapidly worldwide. The associated inflammatory state is a major risk factor for developing type 2 diabetes, cardiovascular diseases, and sleep apnoea, putting immense pressure on global healthcare systems. Abnormal accumulation or dysfunction of adipose tissue can lead to obesity, which is a major risk factor for metabolic and cardiovascular diseases. The mitochondrial unfolded protein response (UPRmt) serves as a critical adaptive mechanism that safeguards cellular homoeostasis during mitochondrial proteostatic stress by orchestrating the expression of chaperones, proteases, and metabolic regulators to restore protein folding capacity and mitigate organelle dysfunction. This review discusses the role of UPRmt in adipocytes, a key player in maintaining metabolic homoeostasis and thermogenesis. Understanding UPRmt’s mechanisms could offer novel therapeutic strategies to combat obesity and its complications.

## 1. Introduction

As a global health challenge, obesity is fundamentally characterized by excessive accumulation and functional dysfunction of adipose tissue. As functional units of adipose tissue, adipocytes maintain systemic metabolic homoeostasis through energy storage, hormone secretion, and thermoregulation. Mammalian adipose tissue primarily consists of white adipose tissue (WAT), brown adipose tissue (BAT), and beige adipose tissue (also termed recruited brown adipose tissue), which exhibits partial brown adipose tissue functions. WAT specializes in energy storage and endocrine functions, while BAT mediates non-shivering thermogenesis and regulates glucose and lipid metabolism through the expression of uncoupling protein 1 (UCP1). Adipose tissue dysfunction – particularly mitochondrial impairment – constitutes a core pathological basis in the pathogenesis of obesity and its associated metabolic disorders, such as type 2 diabetes and cardiovascular disease [1–3]. Adipose tissue dysfunction – particularly mitochondrial impairment – underlies the pathogenesis of obesity and related metabolic disorders such as type 2 diabetes and cardiovascular disease.

Mitochondria, as key organelles within adipocytes, not only sustain fundamental cellular activities but also determine specialized functions such as thermogenesis and lipid metabolism, making them crucial for maintaining the functional integrity of fat cells. As ubiquitous double-membrane organelles in eukaryotes, they convert substrates like glucose and fatty acids into ATP through oxidative phosphorylation – a process accompanied by reactive oxygen species (ROS) production. Mitochondrial dysfunction disrupts metabolic balance, leading to excessive ROS production and impaired synthesis of key molecules like haem and nucleotides, thereby compromising cellular health and inducing disease. Under metabolic or oxidative stress conditions, mitochondria sustain continuous damage, necessitating a sophisticated mitochondrial quality control (MQC) system. This network maintains a healthy mitochondrial pool by integrating mitochondrial biogenesis, fission and fusion, the mitochondrial unfolded protein response (UPRmt), and mitochondrial autophagy. Through protein repair, organelle degradation, inter-organelle communication, and redox homoeostasis regulation, MQC preserves functional integrity and supports adipocyte physiology [4,5].

Imbalances in mitochondrial substrates – such as carbohydrates, fats, or proteins – induce mitochondrial dysfunction, impairing oxidative respiration and energy production [6]. In adipocytes, MQC is critical for functional maintenance; disruption of any MQC component may compromise mitochondrial integrity and adversely affect adipocyte functions. Unlike the endoplasmic reticulum unfolded protein response (UPRer), which primarily alleviates stress by reducing the synthetic load of newly synthesized proteins in the endoplasmic reticulum, UPRmt initiates mitochondrial molecular chaperone and protease expression to mitigate protein toxicity stress by sensing misfolded protein accumulation and activating nuclear transcription programmes. Given the UPRmt’s central role in mitochondrial protection and adipocyte metabolism, elucidating its mechanisms may offer novel therapeutic strategies for obesity-related metabolic disorders.

Current obesity management encompasses pharmacological interventions, endoscopic procedures, and bariatric surgery, each with distinct advantages and limitations. Pharmacological treatments (such as GLP-1 receptor agonists and multi-target agonists) achieve moderate weight loss and metabolic improvements but require long-term use, entail high costs, and carry risks of weight regain and side effects. Endoscopic techniques are minimally invasive and reversible, yet offer limited efficacy with shorter-lasting effects. Bariatric surgery remains the most effective option, delivering lasting metabolic improvements, but carries significant surgical risks, nutritional requirements, and costs [7]. However, whether these interventions directly modulate mitochondrial proteostasis in adipocytes remains largely unexplored. Investigating mitochondrial energy regulation mechanisms through UPRmt and related pathways may reveal novel therapeutic targets. This article reviews recent advances in our understanding of the UPRmt and associated pathways in adipocytes, offering novel research directions for addressing obesity and other metabolic disorders.

## 2. Characteristics and functions of adipocytes

Mammalian adipose tissue is categorized into WAT and BAT, each exhibiting distinct anatomical distribution patterns. White adipose tissue is further subdivided into visceral adipose tissue (VAT) and subcutaneous adipose tissue (SAT). In the human body, visceral adipose tissue is distributed within the greater omentum, mesentery, and abdominal organs, while subcutaneous adipose tissue primarily accumulates beneath the skin, particularly concentrated in the waist, buttocks, and thigh regions [8]. Brown adipose tissue can be categorized based on its origin into congenital brown adipose tissue and inducible brown adipose tissue (beige adipose tissue). When white adipocytes are stimulated by factors such as cold exposure or adrenergic signalling, they undergo a process called ‘browning’, ultimately forming beige adipocytes that predominantly reside within white adipose tissue [3]. Understanding the unique characteristics of each adipocyte subtype under physiological and pathophysiological conditions is crucial for elucidating their roles in metabolic health and disease.

### 2.1. White adipocytes

#### 2.1.1. Physiological condition

White adipocytes exhibit a single-cavity morphology, with their cytoplasm containing a single large lipid droplet occupying most of the space, surrounded by a relatively sparse distribution of mitochondria [8,9]. Anatomically, these cells constitute the WAT distributed throughout the body, comprising specific fat depots: visceral adipose tissue (VAT, located around visceral organs such as the greater omentum, mesentery, and perirenal regions) and subcutaneous adipose tissue (SAT, situated beneath the skin, particularly prominent in the buttocks, thighs, and abdominal areas) [8]. Their primary function is to store energy in the form of triglycerides, serving as the body’s main reservoir for excess calories. Beyond energy storage, white adipocytes exert key endocrine functions by secreting multiple adipokines – including leptin, adiponectin, and adiponectin – which regulate appetite, insulin sensitivity, and systemic metabolism [9]. They also provide mechanical cushioning and thermal insulation, with distinct functional specificities across different fat storage sites: subcutaneous fat is typically associated with beneficial metabolic effects, while visceral fat exhibits higher metabolic activity and contributes more significantly to endocrine signalling [8,9].

#### 2.1.2. Pathophysiological conditions (obesity and metabolic diseases)

Under obese conditions, white adipocytes undergo pathological changes, including hypertrophy (enlargement of existing fat cells) and hyperplasia (increase in the number of fat cells). However, the expansion capacity of white adipose tissue gradually becomes limited, leading to adipocyte dysfunction, hypoxia, and chronic low-grade inflammation. This dysfunction manifests as altered adipokine secretion (e.g. reduced adiponectin, increased leptin and pro-inflammatory cytokines), macrophage infiltration in adipose tissue, and ultimately induces insulin resistance. When subcutaneous WAT storage capacity becomes overloaded, lipids are ectopically deposited in visceral organs such as the liver, muscles, and pancreas. This phenomenon, termed lipotoxicity, exacerbates systemic metabolic dysfunction. The ageing process further promotes the redistribution of fat from subcutaneous to visceral storage depots, thereby intensifying chronic inflammation and metabolic disorders [10].

### 2.2. Brown adipocytes

#### 2.2.1. Physiological characteristics

Brown adipocytes exhibit a multinucleated morphology characterized by numerous microvesicles and a central nucleus. Most notably, they contain abundant mitochondria with densely arranged cristae structures, reflecting their potent oxidative capacity. Anatomically, classic brown adipose tissue in rodents and human infants is distributed in the interscapular, perirenal, and cervical regions. In adult humans, metabolically active brown fat diminishes with age but remains detectable in the supraclavicular, paravertebral, and mediastinal areas. The defining characteristic of brown adipocytes is their thermogenic capacity, mediated by uncoupling protein 1 (UCP1). This protein, localized to the inner mitochondrial membrane, decouples oxidative phosphorylation from ATP synthesis, releasing energy as heat. This process is crucial for non-shivering thermogenesis and cold adaptation. Additionally, brown adipocytes possess secretory functions, releasing brown adipocyte-specific cytokines such as fibroblast growth factor 21 (FGF21), interleukin-6 (IL-6), and transforming growth factor-β2 (TGF-β2). Through autocrine, paracrine, and endocrine actions, these cytokines enhance systemic insulin sensitivity and energy metabolism [2,11–13].

#### 2.2.2. Pathophysiological states (obesity and metabolic disorders)

In obesity, brown adipocyte function is severely impaired, manifested by decreased UCP1 expression, reduced mitochondrial content, and diminished thermogenic capacity. This ‘whitening’ phenomenon is accompanied by accumulation of unilocular lipid droplets, decreased mitochondrial density, and the development of white adipocyte-like characteristics. Concurrently, impaired BAT thermogenesis reduces plasma triglyceride and glucose clearance, triggering systemic metabolic dysregulation [10–12]. Decreased BAT activity in obesity correlates with diminished sympathetic innervation, abnormal thyroid hormone signalling, and mitochondrial dysfunction. Notably, current understanding of brown adipocyte biology primarily stems from rodent models. Although metabolically active brown fat exists in humans, its functional characteristics and therapeutic potential for obesity require further investigation [14].

### 2.3. Beige/brite adipocytes

#### 2.3.1. Physiological condition

Beige adipocytes (also termed brite, for ‘brown-in-white’) are thermogenic adipocytes formed during the browing of white adipose tissue [3]. Morphologically, beige adipocytes exhibit multiple lipid droplets and increased mitochondrial content upon stimulation, resembling brown adipocytes, though their basal state may resemble white adipocytes. Anatomically, beige adipocytes are scattered throughout white adipose tissue, particularly common in subcutaneous white fat. Their induction mechanisms include chronic cold exposure, beta-adrenergic receptor activation, exercise, and specific hormonal signals. Functionally, beige adipocytes express UCP1 and possess thermogenic capacity, though typically at lower levels than classic brown adipocytes. They exhibit significant plasticity, capable of switching between thermogenic and quiescent states according to environmental demands. Crucially, beige adipocytes derive from distinct developmental lineages compared to classical brown adipocytes, originating from smooth muscle-like progenitor cells or through the transdifferentiation of mature white adipocytes [3].

#### 2.3.2. Pathophysiological conditions (obesity and metabolic diseases)

In obesity, the browning response is significantly impaired, manifested by reduced recruitment of beige adipocytes and decreased UCP1 expression in white adipose tissue. This dysfunction is attributed to chronic low-grade inflammation, abnormal sympathetic signalling, and defects in adipocyte-autonomous mitochondrial biogenesis and function. Conversely, promoting WAT browning has emerged as a promising therapeutic strategy for obesity and associated metabolic disorders, as it enhances energy expenditure, improves glucose homoeostasis, and counteracts diet-induced weight gain. The potential metabolic benefits of stimulating beige adipocyte formation and activation through pharmacological, nutritional, and exercise interventions are currently under active investigation [15,16].

In summary, adipocytes exhibit significant morphological and functional heterogeneity across white, brown, and beige subtypes, each possessing distinct anatomical distribution, mitochondrial content, and metabolic functions. These characteristics undergo dynamic regulation under physiological conditions but become severely disrupted in obesity and metabolic diseases – underscoring the importance of characterizing fat cell-specific and adipose tissue-specific features, which is crucial for developing targeted therapeutic strategies.

## 3. Mitochondrial function in adipocytes

### 3.1. The role of mitochondria in white adipocytes

Mitochondria play a central role in WAT by orchestrating key metabolic processes, including oxidative phosphorylation (OXPHOS), fatty acid β-oxidation, and tricarboxylic acid (TCA) cycle , which collectively regulate energy homoeostasis, adipocyte differentiation, and adipokine secretion [17]. In white adipocytes of healthy mice, mitochondria exhibit robust biogenesis and dynamic remodelling capacity to maintain efficient lipid storage and mobilization through balanced oxidative metabolism and minimal ROS production, while maintaining insulin sensitivity. However, in obese mice, WAT mitochondria become markedly dysfunctional, as evidenced by reduced mitochondrial DNA (mtDNA) content, impaired OXPHOS capacity, and diminished fatty acid oxidation, leading to metabolic disturbances and ectopic lipid accumulation [18]. This mitochondrial impairment is further exacerbated by downregulation of Peroxisome proliferator-activated receptor-γ coactivator-1α (PGC1α) signalling, which disrupts adipogenesis and promotes adipocyte hypertrophy over hyperplasia, while elevated ROS generation triggers chronic low-grade inflammation and insulin resistance [10]. Together, these findings highlight the critical role of mitochondrial fitness in maintaining WAT metabolic health and identify mitochondrial dysfunction as a key driver of obesity-associated metabolic complications.

### 3.2. The role of mitochondria in brown adipocytes

Compared to white adipocytes, brown adipocytes contain more abundant and larger mitochondria, which regulate energy metabolism through diverse mechanisms – including lipid oxidation and mitochondrial dynamics – thereby maintaining cellular homoeostasis [19,20]. Studies have shown that BAT mitochondria under obesity exhibit decreased β-oxidation capacity, kinetic imbalance (reduced fusion), impaired autophagic flow and ROS accumulation, which together lead to increased lipid deposition and decreased insulin sensitivity, highlighting the multidimensional regulatory role of mitochondria in BAT [19]. In addition to the aforementioned regulatory roles in energy regulation and cellular homoeostasis, mitochondria are also the core executors of the thermogenic function of BAT. This particular mode of energy expenditure plays an irreplaceable physiological role in maintaining body temperature and regulating energy homoeostasis [21]. It is worth noting that the above conclusions and related mechanisms are mainly from mouse models, and only a few conclusions have been reported in human-related experiments. Under obesity, both mice and human BAT show impaired mitochondrial function, as evidenced by down-regulation of UCP1 expression, reduced mitochondrial number, and morphological abnormalities (e.g. ‘whitening’), leading to reduced thermogenesis and lipid accumulation [22]. Recent studies have revealed that supplementing mouse diets with certain herbal extracts and natural compounds can enhance mitochondrial function in adipocytes. This promotes the browning of WAT and adaptive thermogenesis in BAT, thereby maintaining metabolic homoeostasis. Consequently, restoring BAT mitochondrial function or inducing white fat browning may represent potential strategies for improving obesity and related metabolic disorders [23].

## 4. Definition, classical pathways and functions of UPRmt

### 4.1. Definition of UPRmt

Mitochondria undergo continuous changes in morphology, size, and location within the cell through processes such as division, fusion, autophagy, and mitochondrial protein hydrolysis. This dynamic process ensures that there are enough healthy mitochondria to meet cellular needs, and it is collectively referred to as MQC [24]. In adipocytes, the MQC system is crucial for maintaining normal mitochondrial function. If any component of this system is compromised, it can lead to mitochondrial dysfunction.

Mitochondrial proteostasis is regulated by chaperone proteins and intrinsic proteases, which are essential for maintaining mitochondrial quality control. Among the various strategies involved in preserving mitochondrial proteostasis, the UPRmt is currently a prominent area of research.

UPRmt in mammals is a stress response signalling pathway dedicated to maintaining protein homoeostasis within mitochondria. This pathway is activated when multiple mitochondrial dysfunctions occur – particularly the accumulation of unfolded or misfolded proteins within the mitochondrial matrix. This triggers a reverse signalling cascade from mitochondria to the nucleus, upregulating the transcription of nuclear genes encoding mitochondrial chaperones, proteases, and other protective factors. The following table provides a concise overview of UPRmt triggers, signalling, sensors, transcription factors, and effector proteins (Table 1).

**Table 1.** Overview of the mammalian mitochondrial unfolded protein response (UPRmt) with specific Reference tracing.

Category	Key Component/Name	Primary Function/Description	References
Definition	UPRmt	A retrograde stress-responsive signaling pathway activated by mitochondrial dysfunction, particularly the accumulation of unfolded/misfolded proteins in the matrix. It upregulates nuclear genes to restore mitochondrial proteostasis and function.	[25–27]
Triggers	Misfolded Protein Accumulation	Overexpression of misfolded matrix proteins (e.g. ΔOTC) overwhelms protein folding capacity.	[26,28]
	mtDNA Depletion/Mutation	Loss of mtDNA (e.g. via ethidium bromide) or mutations cause mitonuclear protein imbalance and disrupt OXPHOS assembly.	[26,28,29]
	OXPHOS Disruption	Inhibition of ETC complexes (e.g. by rotenone or oligomycin) impairs mitochondrial proteostasis.	[28,29]
	Import/Translation Inhibition	Blocking protein import (TIM/TOM) or mitochondrial translation (e.g. with doxycycline) leads to unassembled subunits.	[28,29]
	ROS	Elevated mitochondrial ROS can act as a signaling molecule for UPRmt initiation.	[28]
Signaling And Sensors	Mitochondrial Import Efficiency	The competition for import machinery acts as a sensor; compromised import during stress prevents degradation of transcription factors.	[28,29]
	DELE1-OMA1 Pathway	OMA1 protease cleaves DELE1 under stress, releasing a fragment that activates the Integrated Stress Response (ISR) in the cytosol.	[30]
Transcription Factors	ATF5	Functional ortholog of*C. elegans*ATFS-1. Reduced mitochondrial import during stress leads to nuclear accumulation and activation of chaperone/protease genes.	[28,29,31]
	ATF4	Master regulator of the ISR; essential for upregulating a broad set of stress adaptation genes, including UPRmt components.	[29,32]
	CHOP(C/EBP-homologous protein)	bZIP transcription factor induced downstream of eIF2α phosphorylation; activates UPRmt markers and promotes ATF5 expression.	[26,29,33]
Effector Proteins	HSP60/HSP10 (Chaperones)	Classic UPRmt markers; form a chaperonin complex that facilitates proper protein folding in the mitochondrial matrix.	[25,29]
	mtHSP70(Chaperone)	Assists in protein import and folding within the matrix.	[25]
	LONP1 (Protease)	AAA+ protease in the matrix that degrades oxidatively damaged and misfolded proteins.	[28,29,34]
	ClPP (Protease)	Forms a proteolytic complex with ClpX; crucial for degrading damaged proteins and maintaining matrix proteostasis.	[28,29]

### 4.2. Three UPRmt classical pathways

**Figure 1.**
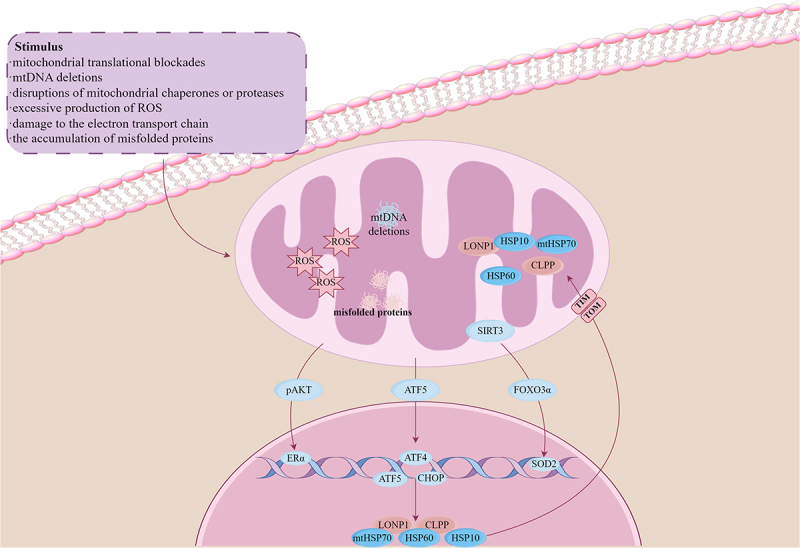
The classic pathways and stimuli of UPRmt (illustrated by ATF5/ATF4-CHOP axis). ROS,Reactive oxygen species; LONP1,Lon peptidase 1; HSP10(HSPE), heat shock protein 10; HSP60(HSPD1), heat shock protein 60; mtHSO70(HSPa),mitochondrial heat shock protein 10; CLPP,Caseinolytic mitochondrial matrix peptidase proteolytic subunit (by figdraw).

Current understanding suggests that multiple signalling pathways activate UPRmt in mammalian cells. In addition to the extensively studied classical activating transcription factor 4/activating transcription factor 5-C/EBP homologous protein axis(ATF4/ATF5-CHOP axis) [35], other significant pathways include the oestrogen receptor α- nuclear respiratory factor 1- high-temperature requirement A2 (ERα-NRF1-HTRA2) pathway and the sirtuin 3- forkhead box O3A- superoxide dismutase 2 (SIRT3-FOXO3A-SOD2) pathway [36,37] (Figure 1). Once mitochondrial proteotoxicity reaches a critical threshold, activation of UPRmt in mammals promotes the expression of mitochondrial chaperone proteins, including heat shock protein 10 (HSP10), mitochondrial heat shock protein 70 (mtHSP70), and heat shock protein 60 (HSP60), as well as protease genes such as Caseinolytic protease P (ClPP) and Lon peptidase 1 (LONP1) [38,39]. These proteins work by mediating the assembly and folding of other proteins and preventing aggregate formation [36]. Notably, some signalling pathways that activate UPRmt do so independently of promoting proteases and chaperone proteins, highlighting the complexity of UPRmt mechanisms in mammals.

#### 4.2.1. ATF4/ATF5-CHOP axis

The most extensively studied classical UPRmt pathway in mammals is the ATF4/ATF5-CHOP axis. When mammalian mitochondria experience stress – triggered by various stimuli, including mitochondrial protein misfolding – ISR is first activated. This activation promotes the phosphorylation of eIF2α, facilitating the action of protein kinase RNA-like endoplasmic reticulum kinase (PERK) and glucose-regulated protein 78 (GRP78). As a result, total protein synthesis decreases while the transcription and translation of key transcription factors such as ATF4, CHOP, and ATF5 increase. Notably, ATF5 can be induced by ATF4 and CHOP and functions through reverse regulation from mitochondria to the nucleus [31]. This cascade ultimately leads to the expression of UPRmt-related protein [40]. Interestingly, many mitochondrial stresses that do not involve protein folding errors – such as those impairing mitochondrial membrane potential, import, or translation – rapidly induce CHOP, generating a response that does not trigger chaperonin activation [32].

#### 4.2.2. SIRT3-FOXO3a-SOD2 axis

The SIRT3-FOXO3a-SOD2 axis, primarily located in the mitochondrial matrix, plays a crucial antioxidant role. This axis is mainly induced by ROS, and studies have shown that it can activate UPRmt, regardless of whether ROS is associated with mitochondrial protein misfolding. Through this activation, the SIRT3-FOXO3a-SOD2 axis contributes to the antioxidant response [36]. The key component of the SIRT3-FOXO3a-SOD2 axis, the SIRT family , belongs to the class III histone deacetylase family and is a highly conserved homolog of the yeast Sir2 protein. This family comprises seven proteins (SIRT1-SIRT7) and is involved in various biological processes, including metabolism, DNA repair, stress response, apoptosis, tumorigenesis, and cellular senescence [41]. Among the SIRT family, SIRT3 has been identified as a key factor in activating UPRmt, exerting antioxidant effects by regulating the activity and localization of the transcription factor forkhead box protein O3a (FOXO3a). Mitochondrial ROS are the primary inducers of this process. As ROS concentrations increase, SIRT3 levels rise, enhancing the deacetylation of FOXO3a. FOXO3a then targets both mitochondria and the nucleus to regulate the activity and localization of SOD2 and catalase synthesis. This promotes the conversion of superoxide to hydrogen peroxide, which is ultimately transformed into water by catalase, thereby exerting antioxidant effects [36,42]. Notably, regulating the expression level of SIRT3 had no significant impact on the expression of key UPRmt-related genes, such as CHOP and LONP1. This suggests that the SIRT3-FOXO3a-SOD2 axis operates independently of the ATF4/ATF5-CHOP axis and does not function through proteases and chaperone proteins [36].

#### 4.2.3. ERα-NRF1-HTRA2/proteasome axis

The mitochondrial intermembrane space (IMS) serves as a crucial transport hub for mitochondrial function and contains various proteins that facilitate these processes. Although the IMS has fewer proteases and lacks heat shock proteins, it remains highly sensitive to misfolded proteins. The ERα-NRF1-HTRA2/proteasome axis has been identified as a vital quality control mechanism for the IMS [37]. Mitochondrial quality control mechanisms primarily involve the regulation of the expression levels of the 26S proteasome and HTRA2. When stress occurs in the IMS, levels of ROS and phosphorylation of protein kinase B (AKT) increase, leading to enhanced activity of ERα. This, in turn, elevates the expression of the mitochondrial regulator NRF1. NRF1 has been shown to increase the expression of HTRA2 and the activity of the 26S proteasome. The upregulation of HTRA2 and the 26S proteasome facilitates the degradation of misfolded proteins [37]. It is important to note that the expression level of the ERα-NRF1-HTRA2/proteasome axis is lower in males compared to females due to the influence of gender on ERα expression. Consequently, the protective effect of the ERα-NRF1-HTRA2/proteasome axis on mitochondria may be more pronounced in females than in males [43].

#### 4.2.4. Interaction between different axes of UPRmt

The three axes of UPRmt mentioned above work synergistically within the organism to mitigate proteotoxicity and oxidative stress damage in the mitochondria [44]. It has been observed that the ATF4/ATF5-CHOP axis can play a compensatory role in maintaining mitochondrial integrity when the ERα-NRF1-HTRA2/proteasome axis is inhibited [43]. For example, in reducing mitochondrial proteotoxicity, there is a synergistic effect between the SIRT3-FOXO3a-SOD2 axis and the ERα-NRF1-HTRA2/proteasome axis. This synergy primarily occurs through the inhibition of the SIRT3-FOXO3a-SOD2 axis by the binding of peroxisome proliferator-activated receptor gamma coactivator 1α (PGC1α), which activates NRF1. Currently, there is significant potential for further research into UPRmt-related mechanisms in mammals.

### 4.3. Interactions between UPRmt and other mitochondrial quality control mechanisms

UPRmt interacts closely with other mitochondrial quality control mechanisms under various physiological and pathological conditions. In mammals, ROS integrates multiple stress signals – including endoplasmic reticulum stress and amino acid starvation – through the eIF2α-ATF4/ATF5 pathway, directly or indirectly regulating the initiation of UPRmt [5].

Significant interactions exist between UPRmt and the endoplasmic reticulum unfolded protein response (UPRer,another crucial mitochondrial quality control mechanism), primarily mediated through three key mechanisms [45]: First, the eIF2α-ATF4/ATF5 signalling axis serves as a crucial molecular bridge; second, mitochondria-associated membranes promote functional coordination via calcium ion flux and ROS signalling; third, metabolic disorders such as type 2 diabetes synergistically activate both pathways through ATF4-dependent mechanisms.

The unfolded protein response activated by misdirected target proteins (UPRam) is a cytoplasmic stress response pathway triggered by the accumulation of precursor proteins that are mislocalized or fail to be successfully imported into mitochondria. Initially discovered in lower eukaryotes, UPRam alleviates protein toxicity stress by upregulating proteasome activity to degrade excess cytoplasmic proteins. Although its homologous mechanism in mammals remains unclear, the activation of the proteasome during mitochondrial protein import dysfunction suggests the evolutionary conservation of this pathway [46]. UPRmt and UPRam are interconnected through their synergistic maintenance of mitochondrial protein homoeostasis. Their interaction primarily occurs in two scenarios: First, when impaired protein import leads to the accumulation of precursor proteins in the cytoplasm (activating UPRam) while simultaneously disrupting mitochondrial protein homoeostasis (activating UPRmt), the retrograde transport of mitochondrial intermembrane space proteins into the cytoplasm may further link these two pathways [47]. Additionally, when mitochondrial translation defects (such as drug inhibition or ribosomal protein mutations) occur, unassembled OXPHOS subunits are produced, activating UPRmt; simultaneously, nuclear-encoded mitochondrial proteins may be mislocalized to the cytoplasm, thereby triggering UPRam [48]. In summary, UPRmt and UPRam jointly respond to protein homoeostasis stress caused by mitochondrial biogenesis abnormalities, and their interaction is particularly important during metabolic stress, input defects, or OXPHOS assembly failure.

### 4.4. Coupling mitochondrial proteotoxic stress to the cytosolic integrated stress response (ISR)

Recent research advances have significantly expanded our understanding of mitochondrial stress signalling in mammals, particularly the intricate cross-regulatory mechanisms between mitochondrial protein toxicity stress and the cytosolic integration stress response (ISR). A key mechanism involves the overlapping activity with the M-AAA protease 1- death life executioner-1- haem-regulated inhibitor (OMA1-DELE1-HRI) pathway: mitochondrial dysfunction activates the inner membrane protease OMA1, which cleaves the intermembrane space protein DELE1 into short fragments (DELE1s) and transports them into the cytoplasm. This fragment binds to and activates the HRI kinase in the cytoplasm, which subsequently phosphorylates eukaryotic initiation factor 2α (eIF2α), ultimately activating ISR transcription factors ATF4, ATF5, and CHOP [49,50]. This signalling axis coordinates transcriptional programmes aimed at restoring mitochondrial protein homoeostasis by upregulating chaperones and proteases while suppressing overall protein synthesis. Concurrently, impaired mitochondrial protein import activates the mammalian UPRmt via the transcription factor ATF5—a functional homolog of C. elegans activating transcription factor associated with stress-1 (ATFS-1) – which shuttles from damaged mitochondria to the nucleus to induce protective gene expression [49,50]. These pathways are tightly coupled with mitochondrial autophagy: persistent mitochondrial damage activates the PTEN-induced kinase 1/parkin (PINK1/Parkin) pathway to clear dysfunctional organelles [49]. Collectively, these findings establish the ISR as a central hub for sensing diverse mitochondrial stresses and coordinating adaptive responses, providing crucial insights into mitochondrial dysfunction in metabolic diseases, including adipocyte dysfunction in obesity [51].

### 4.5. The universal role of UPRmt

As described above, the UPRmt is a conserved stress response pathway that is critical for cellular health across organisms ranging from nematodes to mammals. Its primary function is to maintain mitochondrial protein homoeostasis and overall function, playing a particularly crucial role under stress conditions. The following section briefly outlines the non-cell-specific functions of the UPRmt [29,52].

#### 4.5.1. UPRmt integration of stress signals and maintains mitochondrial homeostasis

Mitochondria contain multiple proteins, most of which originate from the cell nucleus and are transported into mitochondria via the translocase of the outer membrane/translocase of the inner membrane(TOM/TIM) complex. This import process, coupled with ROS inevitably generated during oxidative phosphorylation, challenges mitochondrial protein homoeostasis, necessitating UPRmt with its precise regulatory capacity. This response is activated by multiple stimuli – including mitochondrial translation suppression, mtDNA damage, electron transport chain dysfunction, abnormal chaperone/protease localization, and misfolded protein accumulation – all of which disrupt protein folding and impair function [53]. These stressors can place mitochondria under strain, thereby triggering the activation of UPRmt. Once activated, UPRmt initiates a transcriptional programme. This programme enhances protein folding capacity. It increases protease activity and improves mitochondrial proteostasis. The response ultimately restores organellar function.Through these mechanisms, UPRmt preserves mitochondrial integrity. It ensures cellular survival under stress conditions. This pathway is therefore essential for maintaining mitochondrial homoeostasis.

#### 4.5.2. UPRmt senses mitochondrial protein import

As mentioned above, mitochondrial protein transport is an extremely important process. It has been found that impairments of mitochondrial protein import activate MQC, which maintains overall mitochondrial function and thus cell survival [54]. When mitochondrial protein import is disrupted, unimported mitochondrial precursor proteins overaccumulate in the cytoplasm, a phenomenon known as the mitochondrial precursor overaccumulation stress (mPOS) response, which can stimulate UPRmt [55]. In human cells, the process of mitochondrial protein misfolding signals being transmitted back to the nucleus releases two signals: mtROS and the accumulation of cytosolic mitochondrial precursor proteins (c-mtProt) in the cytoplasm. Upon translocation to the cytoplasm, mitochondrial reactive oxygen species (mtROS) oxidizes DNAJA1 (DnaJ heat shock protein family (Hsp40) member A1), thereby facilitating the recruitment of heat shock protein 70 (HSP70) to c-mtProt. This interaction promotes the release of heat shock factor 1 (HSF1) from HSP70, allowing HSF1 to translocate to the nucleus and activate the transcription of UPRmt-related genes [56]. Through these mechanisms, mitochondrial stress signalling and mitochondrial proteostasis are effectively integrated, initiating the mitochondrial response process.Thus, UPRmt, which regulates mitochondrial protein homoeostasis, is important for maintaining mitochondrial protein import.

#### 4.5.3. The biphasic role of moderate UPRmt in organismal health and longevity

A substantial body of evidence from studies spanning Caenorhabditis elegans, Drosophila melanogaster, and mammalian systems has established that moderate, transient activation of UPRmt confers multiple benefits for overall health and longevity, whereas excessive or persistent activation, as well as complete loss-of-function, can precipitate pathological consequences [53,57].

##### 4.5.3.1. The hormetic window of UPRmt activation: balancing optimal intensity and duration

The mitochondrial hormesis theory posits that mild mitochondrial stress activates enduring cellular protective mechanisms, thereby enhancing stress resistance and promoting longevity [58]. In Caenorhabditis elegans, the level of UPRmt activation positively correlates with lifespan extension achieved through various genetic interventions. Knocking down different mitochondrial ribosomal proteins (MRP) genes induces varying degrees of UPRmt activation (measured via HSP-6:GFP reporter expression), and activation levels correlate significantly with extended mean lifespan [53]. Crucially, this relationship is not infinitely linear but follows a bell-shaped curve: optimal activation yields maximum benefits, while activation beyond the threshold produces detrimental effects [58]. The developmental timing of UPRmt activation critically determines its lifespan-extending effects. In Caenorhabditis elegans, administering mrps-5 RNAi exclusively during the larval stage extended lifespan by approximately 48%, whereas knockdown initiated in adulthood conferred no longevity benefit [53]. This developmental sensitivity window indicates that UPRmt establishes a persistent metabolic programming mechanism throughout life – a phenomenon also observed in mammalian systems: transient mitochondrial stress during embryonic development can induce enduring mitochondrial adaptive effects [58].

##### 4.5.3.2. Core molecular mechanisms underlying the beneficial effects of UPRmt

Moderate activation of the UPRmt can coordinate transcriptional programmes to maintain mitochondrial metabolic homoeostasis while ensuring normal mitochondrial number and function [57]. This response promotes the repair and regeneration of damaged mitochondria through precise regulation, sustaining the efficient operation of the cellular powerhouse [53]. The transcription factor ATFS-1 plays a central role in this process by promoting chemically controlled complex assembly through synchronous restriction of oxidative phosphorylation transcript accumulation encoded by nuclear and mitochondrial DNA, while simultaneously inducing mitochondrial protein homoeostasis genes [57]. This dual regulation both alleviates substrate burden on stressed mitochondria and prevents accumulation of potentially toxic unassembled oxidative phosphorylation subunits.

In mammals, UPRmt serves as a critical regulator of the innate immune system, rapidly responding to and activating defence mechanisms against potential pathogen infection [53]. Mitochondrial toxins produced by bacterial pathogens such as Pseudomonas aeruginosa – including cyanide – inhibit cytochrome c oxidase. The resulting mitochondrial dysfunction activates the UPRmt via ATFS-1 [57]. Nematodes lacking ATFS-1 exhibit hypersensitivity to infection, while those with persistently activated UPRmt demonstrate enhanced intestinal bacterial clearance, suggesting that moderate activation of this pathway provides protective immunity without inducing excessive inflammation [57].

##### 4.5.3.3. Systemic metabolic integration via mitokine signaling and stem cell regulation

The benefits of the UPRmt extend beyond cellular autonomous mechanisms to achieve systemic metabolic regulation through mitochondrial factor signalling. In mice, activation of UPRmt via tetracycline or the triterpenoid compound CDDO significantly increased expression of UPRmt-associated genes (ClPP, LONP1, HSPD1, TID1) in skeletal muscle, adipose tissue, and liver, accompanied by elevated circulating growth differentiation factor 15(GDF15) levels [53]. GDF15 acts through the brainstem receptor GFRAL-RET to promote anorexia and regulate energy homoeostasis, revealing how mitochondrial stress in peripheral tissues communicates with central regulatory centres to coordinate body metabolism [53]. At the haematopoietic stem cell (HSC) level, the mitochondrial stress response helps balance self-renewal and differentiation, supporting sustained renewal and functional homoeostasis of the haematopoietic system [57]. Quiescent HSCs maintain only a small number of mitochondria with low metabolic activity; excessive mitochondrial biogenesis during proliferation disrupts this delicate equilibrium. The histone deacetylase SIRT7, induced by biogenesis-associated mitochondrial protein folding stress, limits mitochondrial translation by inhibiting NRF1, thereby preserving a pristine protein folding environment and maintaining stem cell ‘youthfulness’ [57]. Age-related decline in SIRT7 expression correlates with haematopoietic stem cell dysfunction and reduced leukopoiesis, indicating that maintaining this UPRmt-mediated checkpoint protects regenerative capacity during ageing [57].

##### 4.5.3.4. Mitigating age-related functional decline and preserving tissue integrity

UPRmt activation delays physiological decline in multiple tissues with advancing age. In Caenorhabditis elegans, mrps-5 RNAi worms retain motility, exhibiting twice the activity of controls at day 13 post-adulthood, with a more pronounced difference by day 20 [53]. This functional preservation correlates with delayed muscle fibre disorganization and sustained pharyngeal pumping frequency, indicating UPRmt safeguards tissue integrity during ageing [53]. The NAD+/sirtuin pathway intertwines with UPRmt to promote healthy ageing. Declining NAD levels in ageing tissues impair mitochondrial function [57]. Pharmacological or genetic elevation of NAD levels activates UPRmt via sirtuin-mediated mechanisms, thereby promoting mitochondrial recovery and extending lifespan [57]. This integrated response coordinates mitochondrial biogenesis with protein quality control, ensuring proper folding and assembly of newly synthesized mitochondrial components.

##### 4.5.3.5. The adaptation-pathology threshold: a double-edged sword in cardiometabolic and neoplastic diseases

While moderate UPRmt activation offers benefits, excessive or sustained activation may trigger widespread degradation of mitochondrial proteins, leading to dysfunction and inducing apoptosis [53]. Studies in cardiomyocytes clearly demonstrate this duality: Oligomycin-induced UPRmt activation prevents ischaemia-reperfusion injury, reduces infarct size, and improves cardiac function; conversely, uncontrolled activation exacerbates mitochondrial damage [59]. The mitochondrial matrix protease ClPP exemplifies this threshold effect – defective ClPP in cardiac tissue can paradoxically alleviate mitochondrial dysfunction under certain conditions, whereas its excessive activation promotes excessive protein cleavage and exacerbates tissue injury [59]. The distinction between adaptive and non-adaptive UPRmt hinges on stress severity and duration. Mild mitochondrial dysfunction induced by partial electron transport chain inhibition activates protective UPRmt and extends lifespan, whereas complete respiratory chain failure leads to bioenergetic collapse and cell death [57]. This threshold is particularly evident in cancer biology: mtDNA states inducing moderate UPRmt promote tumour cell survival and metastasis, whereas severe mtDNA mutations causing mitochondrial dysfunction beyond proliferative capacity fail to sustain tumorigenesis [58].

**Figure 2.**
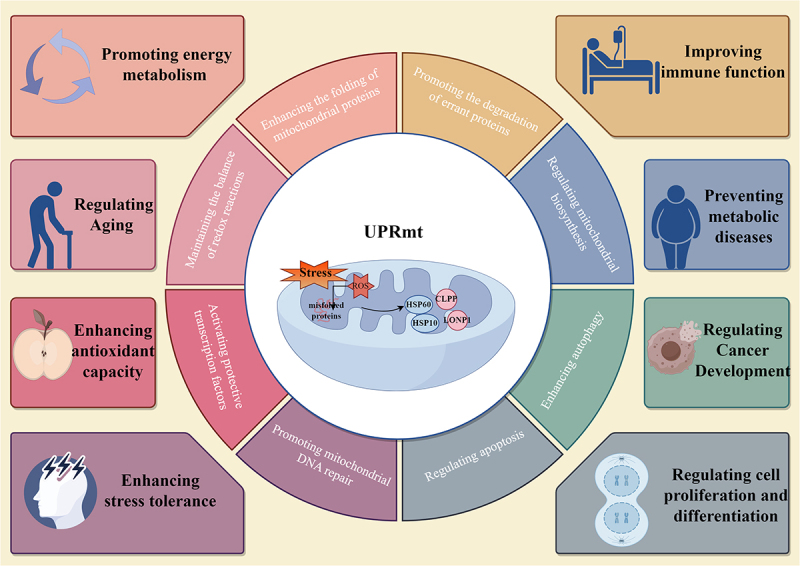
The dual role of UPRmt in health and disease (by figdraw).

In summary, UPRmt possesses multifaceted functions and is often regarded as a double-edged sword (Figure 2). Moderate activation yields multiple benefits: enhancing mitochondrial protein homoeostasis, improving stress tolerance, maintaining stem cell function, delaying age-related decline, and defending against pathogen invasion. However, excessive or sustained activation overwhelms cellular compensatory mechanisms, triggering mitochondrial degradation and apoptosis. Consequently, precisely regulating UPRmt activity within an optimal range has emerged as a major scientific challenge crucial for healthy ageing and the treatment of metabolic disorders, cardiovascular diseases, and tumours.

### 4.6. Physiological and pathological specificity of UPRmt in different adipocyte subtypes

UPRmt exhibits marked specificity across different adipose tissue depots and adipocyte subtypes, reflecting the metabolic functional differences between white, brown, and beige adipocytes. This specificity is determined by mitochondrial abundance, basal metabolic activity, and the unique functional demands of each adipocyte population. Given the current limited research on the direct effects of UPRmt on adipocyte function, this section will focus on key aspects such as the triggers of UPRmt activation, core transcriptional regulators, mitochondrial homoeostasis regulation, and effector proteins, exploring the potential mechanisms and roles through which UPRmt may influence adipocyte function.

#### 4.6.1. UPRmt in white adipocytes: regulating glucose uptake capacity, lipid storage, adipokine secretion, and metabolic health

White adipocytes possess specialized energy storage functions, and their mitochondrial function is closely linked to systemic metabolic homoeostasis. Under physiological conditions, UPRmt helps maintain white adipocyte function, but this response becomes dysregulated during pathological stress states such as obesity.

##### 4.6.1.1. UPRmt correlates with glucose uptake capacity in white adipocytes

Overnutrition induces severe mitochondrial dysfunction in white adipocytes. After 48 hours of exposure to high glucose (25 mM) and/or high free fatty acids (1 mM), 3T3-L1 adipocytes exhibited significantly reduced insulin-stimulated glucose uptake alongside pronounced morphological alterations – reduced mitochondrial volume, denser structure, and distorted cristae architecture [60]. This dysfunction is mediated by altered mitochondrial dynamic protein expression – decreased mitochondrial fusion protein-1 (MFN1) and increased DRP1—alongside downregulation of mitochondrial biogenesis factors including PGC-1α, NRF1, and mitochondrial transcription factor A (mtTFA) [60]. Crucially, these alterations occur without changes in mitochondrial DNA content, indicating a functional rather than quantitative defect. Consequences include increased reactive oxygen species accumulation, loss of mitochondrial membrane potential (ΔΨ), and reduced intracellular calcium levels, forming a vicious cycle of oxidative stress and mitochondrial dysfunction that perpetuates insulin resistance [60]. This demonstrates that the UPRmt, which maintains mitochondrial protein homoeostasis, can influence glucose uptake capacity in white adipocytes to a certain extent.

##### 4.6.1.2. UPRmt influences adiponectin secretion by affecting mitochondrial function

A study linking mitochondrial function to adipocyte endocrine activity revealed that mitochondrial integrity plays a crucial role in adiponectin synthesis [61]. In obese db/db mice, reduced plasma adiponectin levels correlate with diminished mitochondrial content in adipose tissue, both of which are reversed by rosiglitazone treatment. Mechanistically, mitochondrial dysfunction activates a signalling cascade involving endoplasmic reticulum stress, JNK activation, and inducible transcription factor 3 (ATF3), which directly suppresses adiponectin transcription [61]. Pharmacological inhibition of mitochondrial respiratory chain complexes (e.g. rotenone, antimycin A, oligomycin) or mitochondrial protein synthesis (e.g. chloramphenicol) reduces adiponectin secretion, whereas overexpression of nuclear respiratory factor-1 (NRF-1) promotes mitochondrial biogenesis and adiponectin production [61]. This establishes a direct link between mitochondrial protein homoeostasis and secretion of this key insulin-sensitizing adipokine, indirectly demonstrating the importance of UPRmt for adiponectin secretion.

##### 4.6.1.3. Mitochondrial oxidative stress and UPRmt: linking ROS signaling to healthy adipose expansion

The concept of ‘healthy adipose expansion’, contrasting with pathological obesity, hinges critically on mitochondrial redox balance and the stress responses it triggers. UPRmt, serving as a key reverse signalling pathway activated by mitochondrial protein toxicity stress, is frequently initiated by dysregulated ROS production. Using two complementary mouse models – adipose ROS-clearing mice (aP2-Cat/SOD1 double transgenics) and adipose ROS-enhanced mice (Adipoq-Gclc knockout) – research confirms that adipocyte-specific oxidative stress dominates adipose tissue remodelling and systemic insulin sensitivity [62]. Fat ROS-clearing mice exhibited enhanced expansion of subcutaneous and gonadal WAT, characterized by enlarged adipocytes, alongside reduced hepatic ectopic lipid deposition and improved insulin sensitivity. Conversely, fat ROS-enhanced mice displayed restricted adipose tissue expansion, exacerbated hepatic steatosis, and worsened insulin resistance [62]. The underlying mechanism links ROS to mitochondrial and nuclear transcription programmes. ROS suppress lipid synthesis by inhibiting SREBF1 transcriptional activity through reduced protein stability of histone demethylase KDM1A (LSD1) [62]. Such epigenetic and transcriptional alterations may intersect with UPRmt signalling pathway, as both adapt cellular metabolism to mitigate mitochondrial stress. Furthermore, adipose ROS promotes adipose tissue fibrosis (upregulated Col1a1 and Timp1 expression), drives pro-inflammatory M1 macrophage polarization, and suppresses M2 macrophages, thereby limiting healthy adipose tissue expansion capacity [62]. These findings confirm that mitochondrial oxidative stress within adipocytes is a primary determinant of adipose tissue remodelling capacity and metabolic health, with UPRmt potentially serving as a critical link between ROS production, protein homoeostasis, and adipocyte function.

##### 4.6.1.4. ClPP in white adipocytes: a compensatory regulator of mitochondrial mass and insulin sensitivity

The UPRmt-associated protease ClPP plays a unique and tissue-specific role in white adipocyte biology. Mice with complete ClPP knockout (ClPP-/-) exhibit a distinctive phenotype: despite increased food intake, they display reduced adipose tissue mass, improved insulin sensitivity, and enhanced systemic energy expenditure [63]. Notably, this phenomenon is accompanied by white adipose tissue-specific increases in mitochondrial biogenesis, manifested as elevated mitochondrial DNA content (up to 4-fold), upregulation of PGC1α, Tfam, VDAC, and multiple electron transport chain subunits, and enhanced mitochondrial respiration in white adipocytes [63]. This compensatory response is unique to WAT; other tissues such as heart, liver, and brain show no increase in mitochondrial markers. Mechanistically, ClPP deficiency triggers compensatory upregulation of mitochondrial molecular chaperones (HSP60, HSP40, HSP10) and LONP1, likely to stabilize unfolded proteins [63]. The increased mitochondrial mass in WAT directly promotes enhanced respiratory activity, reduced fat mass, and improved insulin signalling (enhanced Akt phosphorylation) [63]. This demonstrates that the absence of components of UPRmt can instead specifically trigger beneficial metabolic adaptations in white adipose tissue.

##### 4.6.1.5. UPRmt in white adipocytes mediates systemic effects via mitokine secretion

A research found that Adipocyte-specific mitochondrial dysfunction activates UPRmt and triggers the secretion of mitokines that coordinate systemic metabolism. Adipocyte-specific Crif1 knockout (AdKO) mice, which exhibit impaired OXPHOS due to defective mitochondrial ribosomal function, display reduced fat mass, increased energy expenditure, and protection from diet-induced obesity [64]. Transcriptomic analysis of white adipose tissue from AdKO mice revealed marked upregulation of GDF15 and FGF21, identifying these as key adipocyte-derived mitokines [64]. GDF15 secreted from adipocytes acts systemically: global deletion of Gdf15 in AdKO mice (AdGKO) abolishes protection against obesity, reduces energy expenditure, and decreases UCP1 expression in white adipose tissue [64]. Furthermore, GDF15 and FGF21 play differential roles – GDF15 regulates energy expenditure and UCP1 induction, whereas FGF21 primarily contributes to glucose homoeostasis and insulin sensitivity [64]. Beyond its systemic metabolic effects, GDF15 also plays a critical role in maintaining adipose tissue immune homoeostasis by regulating macrophage polarization. In AdKO mice, elevated GDF15 promotes M2 macrophage polarization while reducing M1 macrophages, thereby limiting adipose tissue inflammation [65]. This effect is mediated through the IL-4/IL-13 signalling pathway: GDF15 is required for IL-13-induced M2 polarization via the JAK-STAT6 pathway, and GDF15-deficient macrophages fail to undergo M2 polarization even upon IL-4 treatment [65]. Consistent with this, myeloid-specific mitoribosomal defects (LysM-Cre; Crif1 KO) result in reduced GDF15 expression, increased M1 macrophage accumulation in adipose tissue, and systemic insulin resistance [65]. The anti-inflammatory effect of GDF15 is further supported by its ability to increase mitochondrial respiration and fatty acid oxidation in macrophages, thereby promoting an anti-inflammatory phenotype [65]. Collectively, UPRmt activation in white adipocytes, through GDF15 secretion, orchestrates intercellular communication between adipocytes and adipose tissue macrophages to maintain both systemic metabolic homoeostasis and local tissue immune balance [64,65].

#### 4.6.2. UPRmt in brown adipocytes: maintaining thermogenic capacity and preventing whitening

Brown adipocytes are specialized for energy expenditure through UCP1-mediated thermogenesis. Their abundant mitochondrial content renders them highly dependent on mitochondrial protein homoeostasis mechanisms. Consequently, the UPRmt, which regulates mitochondrial homoeostasis, is crucial for their function.

##### 4.6.2.1. LONP1 maintains the thermogenic function of brown adipocytes

LONP1, as a mitochondrial protease, participates in the hydrolytic remodelling of mitochondrial proteins. In visceral adipose tissue, LONP1 expression is upregulated by cold stimulation or β3-adrenergic agonists, promoting adipocyte conversion by selectively degrading specific mitochondrial proteins such as the iron-sulphur subunit B of the succinate dehydrogenase complex. This degradation ensures adequate intracellular succinate levels, which in turn alters the methylation status of thermogenic genomic proteins, promoting the conversion of white adipocytes into beige adipocytes. LONP1 plays a crucial role in regulating adipocyte thermogenesis and maintaining energy homoeostasis, and is also essential for sustaining mitochondrial protein homoeostasis – a mechanism supporting the normal function of adipocytes and other tissues under metabolic stress. Further studies indicate that reduced LONP1 activity in aged mice impairs the conversion of white adipocytes into beige adipocytes, thereby diminishing the body’s capacity to meet thermogenic demands [66]. Reactivating the LONP1-succinate pathway restores beige conversion in aged mouse adipocytes, enhancing their adaptive thermogenic capabilities. In the context of obesity and metabolic syndrome, LONP1 May promote adaptive cellular responses to metabolic changes by regulating mitochondrial protein degradation and synthesis. This regulation indirectly influences glucose metabolism by affecting thermogenesis and energy expenditure in adipocytes. Notably, elevated LONP1 expression in obesity may promote adipocyte browning, increase energy expenditure, reduce glucose storage in adipose tissue, and ultimately improve glucose metabolism [67]. In summary, LONP1 exerts its critical role in lipid metabolism by influencing brown adipocyte differentiation and function.

##### 4.6.2.2. Preventing brown adipose tissue from whitening

A study revealed that in mice with enhanced adipose ROS, ROS promotes lipid accumulation within BAT, downregulates UCP1 and PGC1α expression, and induces characteristic morphological changes indicative of BAT whitening – acquiring a phenotype similar to white adipocytes, such as unilocular lipid droplets. Conversely, mice with low adipose ROS expression maintain the integrity and thermogenic function of brown adipose tissue [62]. This indicates that mitochondrial oxidative stress directly impairs BAT thermogenesis by suppressing mitochondrial function and inducing phenotypic conversion towards white adipose tissue. Indirectly, it suggests that UPRmt activation protects BAT from pathological whitening under metabolic stress conditions.

#### 4.6.3. UPRmt plays a crucial role in maintaining the phenotype of beige adipocytes

UPRmt plays a critical role in directing beige adipocyte formation within white adipose tissue depots upon thermogenic stimulation. LONP1-dependent mitochondrial proteolysis is a prerequisite for this white-to-beige conversion, as demonstrated by MitoTimer reporter mice showing that cold or CL316,243 treatment reversibly increases mitochondrial protein turnover in inguinal WAT(iWAT) [66]. Disruption of LONP1—via siRNA knockdown or adipocyte-specific knockout – impairs cold- or CL316,243-induced beiging. LONP1 Adipocyte-specific Knockout (AKO) mice exhibit attenuated multilocular adipocyte morphology, blunted UCP1 induction, decreased mitochondrial respiration, and lower body temperature upon cold exposure. Lineage tracing with AdipoChaser-LONP1 AKO mice confirms that LONP1 deficiency directly restrains the reprogramming of mature white adipocytes into beige cells.

Mechanistically, LONP1 directs beige adipocyte formation by selectively degrading SDHB. LONP1 physically interacts with SDHB, and its deficiency causes SDHB protein accumulation without altering mRNA levels. Cycloheximide chase and in vitro protease assays confirm direct LONP1-dependent SDHB degradation. This proteolytic regulation ensures adequate intracellular succinate levels during beige adipogenesis, as succinate is markedly decreased in LONP1-deficient adipocytes. Succinate acts as a signalling metabolite by inhibiting α-ketoglutarate-dependent dioxygenases, thereby altering the epigenetic landscape. Cold stimulation increases the succinate/α-KG ratio in iWAT, an effect abolished in LONP1 AKO mice. Chromatin immunoprecipitation sequencing reveals that LONP1 deficiency causes broad decreases in H3K4me1 levels on thermogenic genes (UCP1, COX8B, CIDEA), with succinate treatment restoring these marks and rescuing UCP1 expression. Succinate administration synergizes with β3-adrenergic receptor activation to promote white-to-beige conversion. SDHB overexpression attenuates succinate- and CL316,243-induced beiging, while malonate inhibition of SDH enhances it, confirming the critical role of SDH-mediated succinate metabolism [66]. Adipocyte-specific LONP1 overexpression or dietary succinate supplementation partially restores impaired white-to-beige conversion in aged mice, increasing UCP1 expression and mitochondrial respiration to levels comparable to young controls. Ageing is associated with declining UPRmt function and impaired beige adipocyte formation, with ClPP mRNA decreased in aged human subcutaneous WAT and protein levels reduced by approximately 73% in aged mice [63]. While ClPP deficiency promotes mitochondrial biogenesis specifically in white adipose tissue and improves metabolic health through elevated OPA1 and UCP2 expression, ClPP is not required for mammalian UPRmt induction, demonstrating distinct roles for different UPRmt-associated proteases in adipose tissue biology. These findings identify LONP1-dependent succinate regulation as a key mechanism underlying beige adipocyte formation and suggest that targeting this axis could reinstate thermogenic capacity in ageing [63,66].

#### 4.6.4. Differential UPRmt regulation between BAT and WAT

Brown and white adipose tissues exhibit fundamentally different UPRmt regulation. In MitoTimer reporter mice, which track mitochondrial protein turnover, brown adipose tissue displays constitutively high rates of mitochondrial protein turnover regardless of temperature or β3-adrenergic stimulation, whereas white adipose tissue shows dynamic regulation with increased turnover only upon thermogenic activation [66]. Consistently, LONP1 and CLPP protein levels increase in white adipose tissue (both iWAT and eWAT) following cold exposure or CL316,243 treatment, but remain unchanged in BAT [66]. This suggests that brown adipocytes maintain constitutive UPRmt activity to support their high mitochondrial demand, while white adipocytes induce UPRmt adaptively during thermogenic remodelling. Furthermore, the effects of UPRmt protease deficiency on brown and white adipocytes exhibit significant differences. UCP1-Cre-mediated LONP1 knockout (LONP1f/f; UCP1-Cre) to specifically delete LONP1 in brown adipocytes did not impair cold-induced thermogenesis or brown adipose tissue morphology, indicating that LONP1 in interscapular brown adipose tissue is largely dispensable for adaptive thermogenesis [66]. Conversely, Adipoq-Cre-mediated LONP1 knockout, while affecting both brown and white adipocytes, exhibited a primary phenotype – impaired conversion of white fat to beige fat and reduced systemic energy expenditure – driven predominantly by its impact on white/beige adipocytes rather than classical brown fat cells [66]. This highlights a fundamental difference in UPRmt requirements between brown adipocytes and white adipocytes.

#### 4.6.5. Human relevance: LONP1 expression in visceral adipose tissue associates with metabolic health

The relevance of UPRmt to human adipose tissue biology is supported by studies of LONP1 expression in visceral adipose tissue (VAT). In 48 normoglycemic women undergoing surgery, LONP1 mRNA and protein expression are significantly higher in VAT from overweight or obese individuals (BMI ≥ 23 kg/m^2^) compared to lean individuals [67]. LONP1 expression positively correlates with BMI, and bioinformatics analysis of the Genotype-Tissue Expression (GTEx) database reveals that high LONP1 expression in VAT is associated with upregulation of genes involved in glucose and lipid metabolism, including the citrate cycle, oxidative phosphorylation, insulin signalling, and regulation of lipolysis [67]. Similarly, in BXD recombinant inbred mouse strains, Lonp1 expression in subcutaneous WAT positively correlates with glucose tolerance [67]. These findings suggest that elevated LONP1 in obesity may represent a compensatory homoeostatic mechanism to preserve mitochondrial function and metabolic health, consistent with its role in promoting beige adipocyte formation and thermogenic capacity.

In summary, UPRmt exerts distinct and specialized functions across adipocyte subtypes to regulate metabolic homoeostasis and cellular identity. In white adipocytes, UPRmt maintains systemic metabolism by modulating adipokine secretion (e.g. adiponectin via the ER stress-JNK-ATF3 axis), controlling de novo lipogenesis through the KDM1A-SREBF1 pathway, and orchestrating tissue remodelling via mitokines such as GDF15 and FGF21. Compensatory UPRmt activation, as observed in ClPP deficiency, promotes mitochondrial biogenesis specifically in white adipose tissue and improves systemic metabolism. In brown adipocytes, UPRmt preserves thermogenic capacity by sustaining mitochondrial proteostasis and preventing pathological whitening; notably, LONP1 regulates succinate levels to control epigenetic programming of thermogenic genes, with classical brown adipocytes exhibiting constitutive UPRmt activity distinct from recruitable beige cells. In beige adipocytes, UPRmt is essential for white-to-beige conversion, wherein LONP1-mediated degradation of SDHB ensures adequate succinate levels, which inhibit α-ketoglutarate-dependent dioxygenases to promote H3K4me1 enrichment on thermogenic gene loci. This proteolytic rewiring is required for optimal beige adipogenesis and can be targeted to restore thermogenic capacity during ageing. Collectively, these findings establish UPRmt as a critical determinant of adipocyte identity and function, with therapeutic implications for obesity, insulin resistance, and age-related metabolic decline.

## 5. Pharmacological and lifestyle modulators of adipocyte UPRmt

Contemporary obesity treatment approaches primarily encompass lifestyle interventions, pharmacotherapy, endoscopic procedures, and bariatric surgery, each with distinct mechanisms and efficacy characteristics. Lifestyle modifications, including dietary changes and increased physical activity, remain the foundation of obesity management and can induce 7–10% mean weight loss over 52 weeks [7]. Pharmacological options have expanded considerably, with approved agents including orlistat (a gastric and pancreatic lipase inhibitor), phentermine-topiramate, naltrexone-bupropion, and GLP-1 receptor agonists such as liraglutide and semaglutide [7]; these medications primarily reduce hunger and diminish the rewarding effects of food by acting on central appetite pathways, with most achieving 5–9% weight loss within 12 months, while semaglutide can achieve 10–12% [68,69]. Notably, the once-weekly dual GIP/GLP-1 receptor agonist tirzepatide demonstrated an average weight loss of 20.9% over 72 weeks in obese patients without type 2 diabetes in a Phase III clinical trial [70]. Beyond central appetite suppression, GLP-1 receptor agonists, including exenatide, liraglutide, and tirzepatide, have been shown to induce profound adipose tissue remodelling. Preclinical evidence indicates that GLP-1 R agonism promotes the ‘browning’ of white adipose tissue, characterized by upregulation of UCP-1 and PGC-1α alongside increased mitochondrial biogenesis, thereby enhancing thermogenesis and fatty acid oxidation, effects at least partially mediated through the SIRT1-AMPK signalling axis [71]. Clinically, GLP-1-based therapy significantly reduces total fat mass and optimizes body composition, with fat loss outpacing lean mass reduction [70], while also alleviating obesity-associated inflammation by downregulating pro-inflammatory cytokines such as TNF-α, decreasing circulating high-sensitivity C-reactive protein levels, and improving systemic oxidative stress markers [72,73]. Given that SIRT1 activation is closely associated with enhanced mitochondrial biogenesis and proteostasis, GLP-1 receptor agonists may engage UPRmt as a compensatory mechanism to cope with increased mitochondrial load and oxidative demands, thereby maintaining mitochondrial function and alleviating adipocyte dysfunction during active weight loss [71].

**Figure 3.**
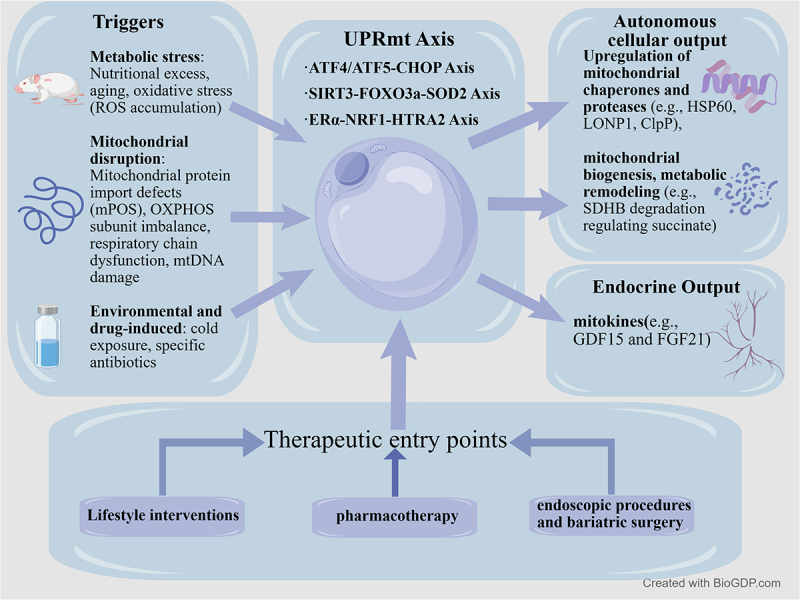
Regulatory network of UPRmt in adipocytes: from stress sensing to systemic metabolism and therapeutic Intervention (Created with BioGDP.com).

Although multiple animal studies mentioned above have confirmed the positive benefits of UPRmt for obesity treatment, direct evidence from human studies regarding the effects of existing drug therapies, lifestyle modifications, and bariatric surgery on mitochondrial protein homoeostasis in adipose tissue and its associated UPRmt nodes remains insufficient. The multi-target agonist tirzepatide activates both GLP-1 and GIP receptors, yet its effects on adipose tissue mitochondrial pathways remain unexplored [70]. Notably, the STEP and SCALE clinical trials of semaglutide and liraglutide focused on weight loss, glycaemic control, and cardiovascular outcomes, without investigating adipose tissue mitochondrial markers or UPRmt activation [74–76]. Similarly, bariatric surgery studies have demonstrated significant metabolic improvements, including remission of type 2 diabetes and reduced cardiovascular events [77–79], yet the contribution of adipose tissue mitochondrial remodelling to these outcomes remains systematically unexplored. Therefore, while GLP-1 agonists and bariatric surgery markedly improve obesity-related metabolic dysfunction, whether these benefits involve direct regulation of adipocyte UPRmt or mitochondrial protein homoeostasis remains an unresolved question. This requires dedicated research evaluating specific experimental indicators in adipose tissue, such as mitochondrial chaperone expression, protease activity, and mitochondrial cytokine secretion (Figure 3).

## 6. Discussion

The UPRmt in adipocytes is not a singular, uniform stress response but exhibits significant cell-type and adipose tissue specificity, acting as a key determinant of metabolic health and cellular identity. Although the underlying UPRmt mechanisms are conserved, their outputs are profoundly influenced by the unique physiological demands and pathological stressors of white, brown, and beige adipocytes. In white adipocytes, UPRmt activation is intrinsically linked to maintaining systemic metabolic homoeostasis, primarily through regulating insulin sensitivity, lipid storage capacity, and the secretion of key adipokines and mitochondrial factors. The induction of mitochondrial cytokines like GDF15 serves as a critical non-autonomous cellular signal, coordinating systemic energy expenditure and glucose metabolism while regulating local intercellular communication by promoting anti-inflammatory M2 macrophage polarization within the adipose tissue microenvironment. Conversely, in thermogenic brown and beige adipocytes, UPRmt is essential for maintaining mitochondrial integrity and identity. A prime example is the LONP1-SDHB-succinate axis, demonstrating how regulated proteolysis directly links mitochondrial metabolism to epigenetic programming, enabling the white-to-beige cell transition essential for adaptive thermogenesis. This reveals a fundamental trade-off: while UPRmt-driven protein homoeostasis is protective, its dysregulation or failure triggers opposite pathologies – such as impaired beige cell recruitment during ageing due to LONP1 decline, or metabolic dysfunction in obesity when oxidative stress overwhelms compensatory mechanisms. Furthermore, the integration of UPRmt with broader cellular stress networks – such as the ISR via the OMA1-DELE1-HRI pathway – highlights the complexity of mammalian mitochondrial surveillance mechanisms, which transcend classical linear signalling cascades. Despite these advances, significant cognitive gaps remain. While mitochondrial stress response activation is a common feature distinguishing healthy adaptation from pathological states in adipose tissue, the precise causal directionality, thresholds differentiating acute adaptive from chronic dysregulated signalling, and roles of various mitochondrial factors in cellular autonomy versus systemic regulation remain unclear. For example, elevated LONP1 expression in visceral fat of obese individuals may represent either a beneficial compensatory response to metabolic stress or a maladaptive signal leading to dysfunction. Notably, these findings hold substantial translational medical significance yet remain under-explored. Whether the marked metabolic improvements observed with modern anti-obesity drugs (such as GLP-1 receptor agonists and multi-effect agonists) or bariatric surgery are partially mediated by direct regulation of adipocyte UPRmt and mitochondrial protein homoeostasis remains a critical unanswered question. It is plausible that these interventions alleviate systemic inflammation and improve systemic metabolism, thereby relieving mitochondrial stress in adipocytes. However, their direct effects on pathways such as the LONP1-succinate axis or adipose tissue GDF15 secretion remain unsystematically investigated. Filling these research gaps requires future studies to overcome the limitations of observational associations. By employing genetic and pharmacological tools in preclinical models and human adipose tissue samples, researchers can establish causality, define activation thresholds, and ultimately determine whether targeted regulation of UPRmt represents a viable therapeutic approach for combating obesity and its associated metabolic diseases.

Future research efforts should be directed towards the following priorities: first, to delineate the precise thresholds of UPRmt activation in distinct adipocyte subtypes and adipose depots, distinguishing acute adaptive responses from chronic maladaptive signalling – a crucial prerequisite for interpreting context-dependent metabolic outcomes; second, to establish standardized and validated in vivo readouts of UPRmt activity across white adipose tissue, brown adipose tissue, and beige adipocyte induction models, thereby facilitating rigorous cross-study comparisons; third, to systematically evaluate whether clinically approved anti-obesity pharmacotherapies – including GLP-1 receptor agonists and multi-agonists – directly modulate UPRmt markers in adipocytes or exert their effects indirectly through systemic mediators such as sympathetic nervous activity, weight loss, or resolution of inflammation; and fourth, to dissect the cell-autonomous versus endocrine functions of mitokines originating from adipocyte mitochondrial stress, elucidating how local perturbations are transduced into systemic metabolic benefits. With a thorough understanding of the aforementioned mechanism, we can expect to precisely regulate adipocyte function by modulating UPRmt, thereby providing safer and more effective therapeutic options for obesity and its associated diseases.

## Data Availability

Data sharing is not applicable to this article as no data were created or analysed in this study.
